# Impact of transcranial direct current stimulation combined with median nerve stimulation on CRS-R in patients with prolonged disorders of consciousness after cerebral hemorrhage: protocol for a randomized controlled trial

**DOI:** 10.3389/fneur.2025.1497316

**Published:** 2025-06-10

**Authors:** Hanbo Chen, Shujuan Huang, Si Chen, Zitian Wang, Yong Luo, Yongliang Guo, Weifeng Wen, Jinfeng Liang, Yunhong Deng, Xiao Lv

**Affiliations:** ^1^Department of Rehabilitation Therapy, Guangdong Sanjiu Brain Hospital, Guangzhou, China; ^2^Zhujiang Hospital, Southern Medical University, Guangzhou, China; ^3^Department of Rehabilitation Medicine, Guangdong Sanjiu Brain Hospital, Guangzhou, China

**Keywords:** transcranial direct current stimulation, median nerve stimulation, prolonged disorders of consciousness, CRS-R, protocol

## Abstract

**Background:**

Prolonged disorders of consciousness (pDoC) resulting from intracerebral hemorrhage (ICH) significantly impair patients’ quality of life. This study aims to investigate the therapeutic effects of transcranial direct current stimulation (tDCS) combined with median nerve stimulation (MNS) in patients with pDoC 3–12 months post-ICH.

**Methods:**

This prospective, double-blind, randomized controlled trial will enroll 138 eligible patients with pDoC following ICH. Participants will be randomly assigned to three groups of 27 each: (1) tDCS + MNS, (2) sham tDCS + MNS, and (3) tDCS + sham MNS. The intervention will last for 4 weeks, followed by a 6-month follow-up period. The primary outcome measure is the change in the Coma Recovery Scale-Revised (CRS-R) scores. Secondary outcomes include electrophysiological and neuroimaging data. Assessments will be conducted at baseline, after 4 weeks of treatment, and at 2 weeks, 4 weeks, 12 weeks, and 24 weeks post-intervention.

**Discussion:**

The strengths of this study include the combined intervention approach of tDCS and MNS, as well as comprehensive outcome measures. This intervention may promote consciousness recovery through multiple mechanisms, including enhanced neuroplasticity and modulation of brain networks. This study aims to provide a novel approach for consciousness-promoting treatment in pDoC, potentially improving patient prognosis and quality of life.

**Clinical trial registration:**

https://www.chictr.org.cn/showproj.html?proj=203618, identifier ChiCTR2300075190.

## Introduction

Prolonged disorders of consciousness (pDoC)refer to a state of impaired consciousness lasting more than 28 days after brain injury, primarily encompassing vegetative state (VS)/unresponsive wakefulness syndrome (UWS) and minimally conscious state (MCS) (1). MCS can be further divided into MCS + and MCS- depending on specific behavioral signs (2), with MCS + patients showing command following, intelligible verbalization, or gestural or verbal yes/no responses, while MCS- patients only show non-reflex movements such as visual pursuit, localization of noxious stimulation, or appropriate affective responses. MCS- is characterized by the absence of language function despite the presence of these non-reflexive behaviors. pDoC can result from various aetiologies, including traumatic brain injury, intracerebral hemorrhage (ICH), ischemia, anoxia, and other mixed causes. In the context of this study, we define consciousness as the clinical measurable capacity for awareness of self and environment, which can be operationalized and assessed through standardized tools like the CRS-R. This operational definition focuses on observable behavioral responses indicating levels of wakefulness and awareness, which serves our clinical research purpose. We acknowledge that this definition may be distinguished from other broader perspectives that explore the wider implications of consciousness for human development and evolution. For instance, Kotchoubey (3) proposed that human consciousness originates at the intersection of communication, play, and tool use. Likewise, Dresp-Langley (4) suggested that consciousness extends beyond neural domains to facilitate adaptation to unprecedented challenges. This study specifically focuses on pDoC following ICH. Patients with pDoC exhibit severe impairments in consciousness level and cognitive function, significantly affecting their quality of life and imposing a substantial burden on families and society (5). Recent epidemiological studies indicate that the incidence of VS/UWS is approximately 5–10% among patients with severe brain injuries (6). The overall incidence of pDoC (including both VS/UWS and MCS) among ICH survivors is estimated to be higher (7). In China, with an estimated 400,000–600,000 new cases of ICH annually, potentially leading to 20,000–60,000 cases of pDoC, research into effective consciousness-promoting methods for pDoC holds significant societal value (8).

Current treatments for pDoC include pharmacological interventions (e.g., amantadine, zolpidem), physical therapy, sensory stimulation, and neuromodulation techniques (9). However, these approaches have limitations: pharmacological treatments often yield inconsistent results with high individual variability and potential severe adverse effects (10); traditional rehabilitation therapies progress slowly with limited efficacy (11); while invasive neuromodulation techniques (such as deep brain stimulation) show promise, they carry higher risks and are not suitable for widespread application (12).

Given the limitations of existing treatments, non-invasive neuromodulation techniques have garnered considerable attention in recent years, particularly the combined application of transcranial direct current stimulation (tDCS) and median nerve stimulation (MNS). tDCS applies weak direct current through scalp electrodes, modulating cortical excitability and promoting neuroplasticity (13). Thibaut et al. (14) found that a single session of tDCS can temporarily improve behavioral responses in MCS patients, while Wu et al. (15) demonstrated that repeated tDCS might enhance consciousness levels in patients with disorders of consciousness (DoC). MNS influences central nervous system function through peripheral nerve stimulation (16). Zhang et al. (17) discovered that MNS can increase electroencephalogram complexity in VS/UWS patients, and Raichur et al. (18) explored the combined application of tDCS and MNS in DoC patients with traumatic brain injury, with preliminary results suggesting that combined therapy may be superior to monotherapy. The combination of these two techniques offers advantages of high safety, ease of operation, and relatively low cost, potentially producing synergistic effects through different mechanisms of action (16).

However, current research on combined tDCS and MNS treatment for pDoC patients has limitations: existing studies have primarily focused on patients with traumatic brain injury, with fewer studies on pDoC patients following ICH (19); most studies have small sample sizes and short follow-up periods, making it difficult to evaluate long-term treatment effects (19, 20). Furthermore, recent literature suggests that the period of 3–12 months post-ICH is critical for neurological recovery, indicating that patients within this time window may have greater potential to respond to neuromodulation therapy (21).

Based on these considerations, we have designed a prospective randomized controlled trial at the Rehabilitation Department of Guangdong Sanjiu Brain Hospital to investigate the therapeutic effects of combined tDCS and MNS in pDoC patients following ICH. This study will focus on patients 3–12 months post-ICH and include a 6-month follow-up period to comprehensively assess the long-term efficacy, safety, and mechanisms of action of this combined treatment approach. This research aims to provide more reliable and comprehensive scientific evidence for the clinical management of pDoC following ICH.

## Study design

This is a prospective, single-center, three-arm parallel randomized controlled trial. Patients will be randomly assigned to three groups: (1) tDCS+MNS group: receiving real tDCS and MNS treatment; (2) Sham tDCS+MNS group: receiving sham tDCS and real MNS treatment; (3) tDCS+Sham MNS group: receiving real tDCS and sham MNS. Each group will consist of 27 patients, with treatment lasting for 4 weeks (22).

Randomization will be implemented using a web-based central randomization system, developed by an independent statistician using the “blockrand” package in R software (version 4.1.0) (23). We will employ a stratified randomization strategy to ensure balanced baseline characteristics across groups. Randomization will be stratified by age (≤50 years/>50 years) and baseline consciousness level (vegetative state/minimally conscious state) (9). The allocation ratio will be 1:1:1, using variable block sizes (4 or 6) to enhance allocation unpredictability. To ensure allocation concealment, the randomization sequence will be stored in the central randomization system, and the study coordinator will only obtain the allocation result through this system after the patient has completed all baseline assessments and confirmed eligibility. The system will send the allocation result to the therapist responsible for implementing the intervention via encrypted email.

This study employs a triple-blind design, where patients, outcome assessors, and data analysts will be blinded to treatment allocation. Patients, outcome assessors, and data analysts will be blinded to treatment allocation. To achieve this, we will adopt the following strategies: (1) All patients will receive identical electrode placement and stimulation duration, regardless of real or sham stimulation. (2) tDCS and MNS devices will be pre-programmed to provide either real or sham stimulation. These devices will be identical in appearance and operated by technicians not involved in patient assessment. (3) Sham stimulation will deliver a brief current for the initial 30 s, mimicking the sensation of real stimulation, before gradually ramping down to zero. This sham tDCS protocol has been validated in previous studies with both healthy participants and patients with disorders of consciousness, showing that participants cannot reliably distinguish between real and sham stimulation (20). (4) All research personnel will receive standardized training to ensure consistency in treatment and assessment procedures. To maintain blinding, we will implement the following measures: (1) All research personnel and participants will be instructed not to discuss treatment details during the study. (2) The effectiveness of blinding will be regularly assessed by asking assessors to guess the type of treatment they evaluated. For patients who regain communication ability during the study (emerging from MCS), we will also ask them to guess which treatment they received, but this will not be possible for patients who remain in pDoC throughout the study. (3) During the data analysis phase, treatment groups will be coded (e.g., A, B, C) until all analyses are completed. Unblinding will only occur in the event of serious adverse events or when patient safety considerations necessitate it. The unblinding procedure will be executed by an independent safety monitoring committee not involved in daily patient management.

Patient recruitment is scheduled to begin on October 1, 2024, with all follow-ups expected to be completed by September 31, 2025. The primary outcome measure (CRS-R scores) will be assessed at baseline, after 4 weeks of treatment, and at 2 weeks, 4 weeks, 12 weeks, and 24 weeks post-intervention (24).

**Figure 1:**
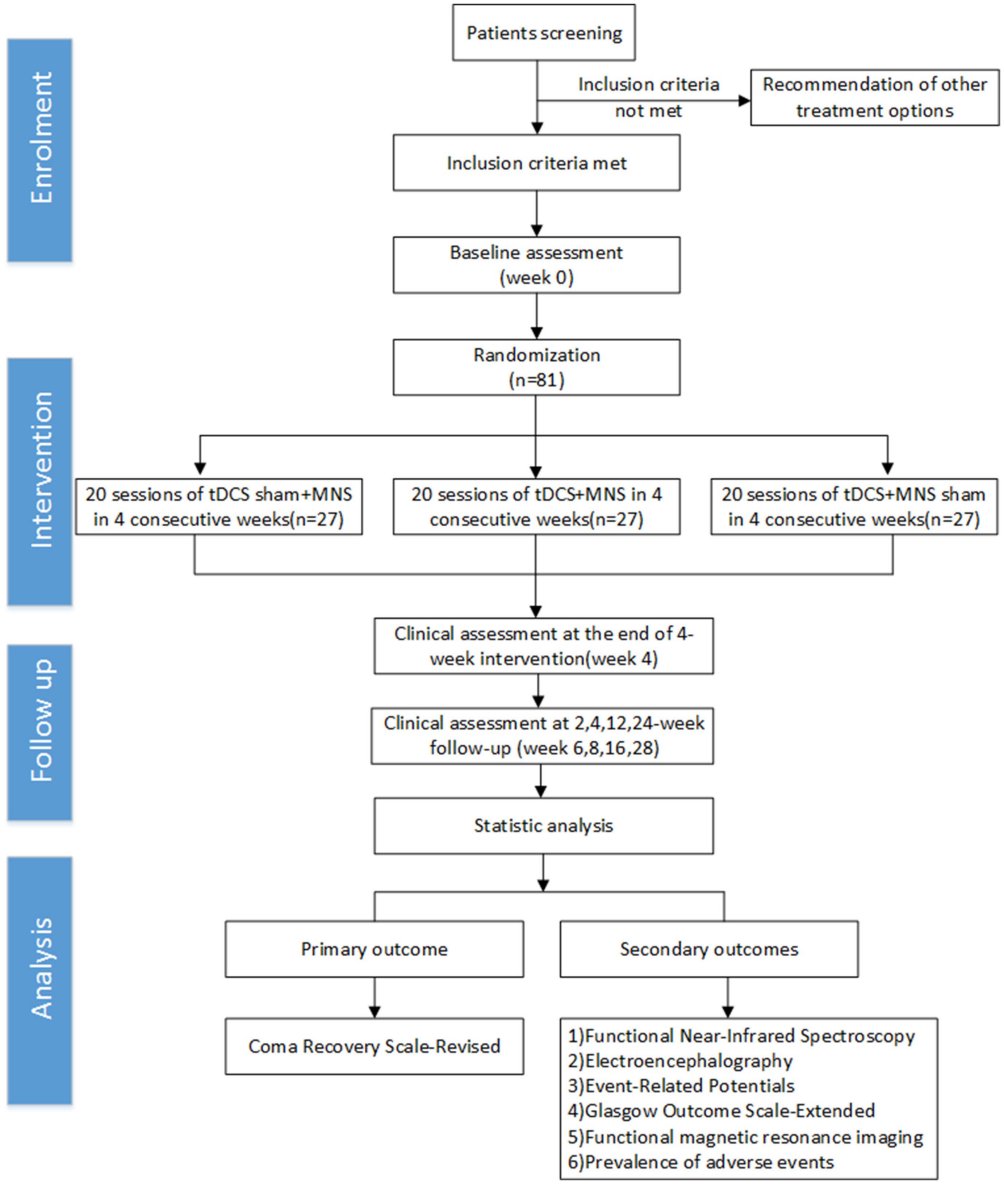
Study design (randomized controlled trials) and assessment time points (consort chart).

All procedures will strictly follow the SPIRIT (Standard Protocol Items: Recommendations for Interventional Trials) guidelines and checklist, as well as the consolidated standards of reporting trials guidelines (Figure 1).

### Study population

This single-center randomized controlled trial will recruit patients with pDoC due to ICH, specifically those in the chronic phase (3–12 months post-injury). The study will be conducted at the Department of Rehabilitation, Guangdong Sanjiu Brain Hospital.

#### Inclusion criteria

(1) Age: 18–65 years; (2) Diagnosed with DOC (vegetative state or minimally conscious state) according to the Coma Recovery Scale-Revised (CRS-R) (24); (3) Time since ICH: 3–12 months, which represents the chronic phase for hemorrhagic etiology; (4) Stable vital signs without need for mechanical ventilation; (5) No significant changes in CRS-R total score (defined as a change of ≤2 points) for at least 2 weeks prior to enrollment; (6) No skull defects or metal implants in the brain that could interfere with tDCS; (7) Written informed consent obtained from the legal representative.

#### Exclusion criteria

(1) Severe complications that may affect assessment or treatment, such as severe spasticity, joint contractures, or pressure ulcers; (2) History of epilepsy or seizures within the past 3 months; (3) Pregnancy or lactation; (4) Participation in other interventional studies within the past 30 days; (5) Contraindications for tDCS or MNS, such as skin lesions at stimulation sites, implanted electronic devices, or allergies to electrode materials; (6) Current use of medications that may significantly alter cortical excitability (e.g., high-dose benzodiazepines, antiepileptic drugs) (13); (7) Neuroimaging evidence of severe brain atrophy (defined as a global cortical atrophy scale score >2 on the 0–3 Pasquier scale, or ventricular-brain ratio >0.25 on axial MRI slices) or hydrocephalus (defined as Evans’ index >0.30 on axial CT or MRI slices). These assessments will be conducted by two independent neuroradiologists with experience in interpreting neuroimaging in patients with disorders of consciousness; (8) History of neurodegenerative diseases or psychiatric disorders; (9) Inability to complete the full course of treatment or follow-up assessments.

### Sample size

Sample size calculation was performed using the Coma Recovery Scale-Revised (CRS-R) score as the primary outcome measure. We employed a one-way analysis of variance (ANOVA) model to determine the required sample size (25). Calculations were conducted using G*Power software (version 3.1.9.7, University of Düsseldorf, Germany) (26).

Based on previous similar studies (14, 27), we assumed a clinically significant difference of 4 points in CRS-R scores between the treatment and control groups. This threshold is based on previous research by Estraneo et al. (24), which demonstrated that a 4-point change in CRS-R score is associated with meaningful transitions between diagnostic categories and correlates with clinically observable improvements in patient responsiveness and function, with a standard deviation of 7.3 points. The significance level (*α*) was set at 0.05, and the power (1-*β*) at 0.8, which are commonly used parameters in clinical trials (28).

For a 3 × 6 mixed repeated measures ANOVA (one 3-level between-subjects factor and one 6-level within-subject factor), G*Power calculated a required sample size of 22 participants per group to detect a medium effect size (*f* = 0.25) with 80% power at a 5% significance level, considering correlations among repeated measures of 0.5 and a nonsphericity correction of 0.75. To account for potential dropouts and loss to follow-up, we increased the sample size by 20% (19, 29). Consequently, the final sample size was determined to be 27 participants per group, totaling 81 participants for the three-group study design.

### Experimental group intervention

**Figure 2:**
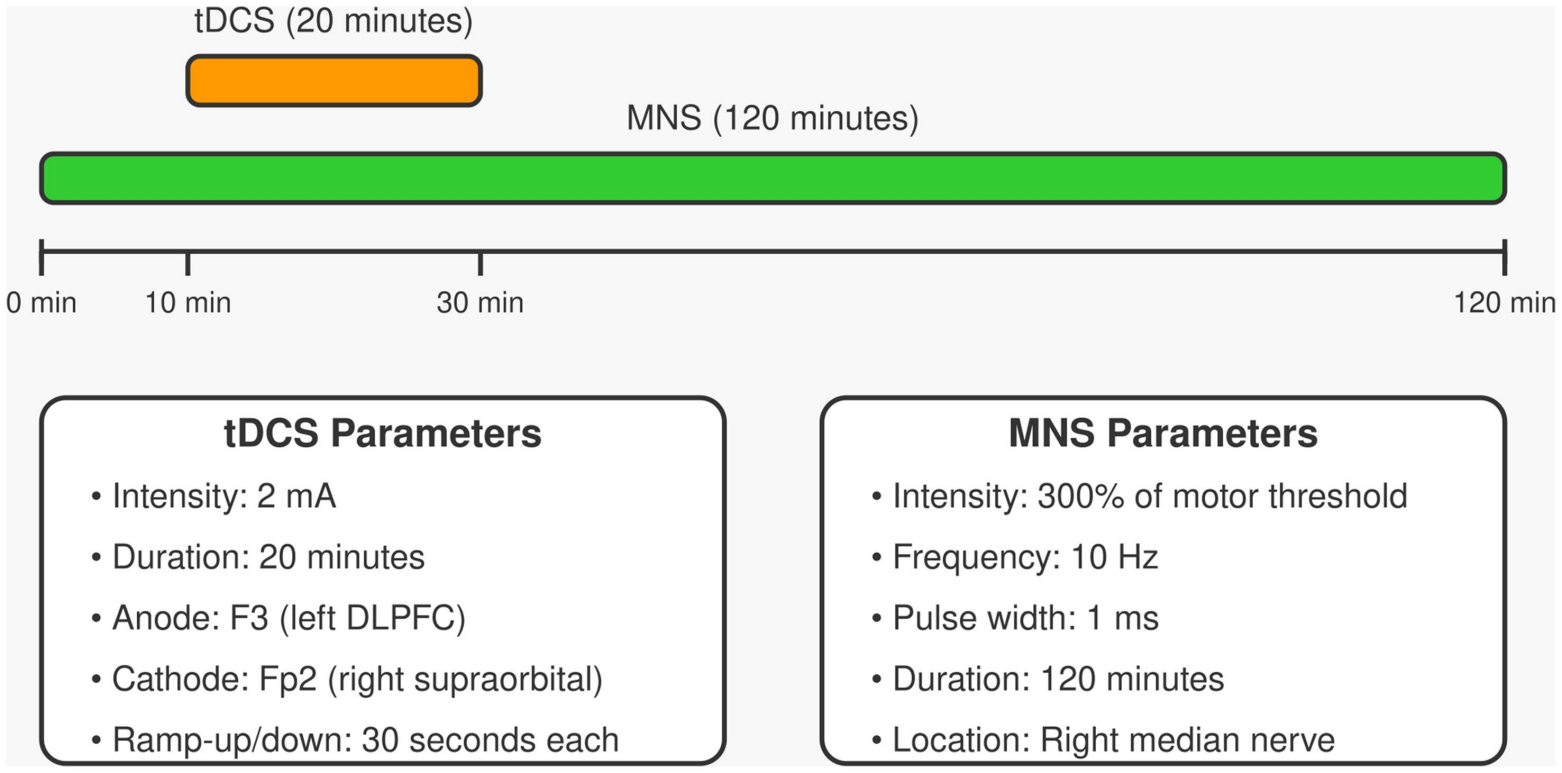
Combined tDCS and MNS protocol for patients with pDoC.

The experimental group (tDCS+MNS group) will receive tDCS combined with MNS. tDCS will be administered using a Starstim tDCS stimulator (Neuroelectrics, Barcelona, Spain). The anode electrode (5 × 7 cm) will be placed over the left dorsolateral prefrontal cortex (F3 position according to the international 10–20 system), and the cathode electrode (5 × 7 cm) will be placed over the right supraorbital area (Fp2 position). Stimulation will be delivered at 2 mA for 20 min, with ramp-up and ramp-down periods of 30 s each, once daily for 5 consecutive days per week, for 4 weeks. MNS will be administered using a Digitimer DS7A constant current stimulator (Digitimer Ltd., Hertfordshire, UK). Electrodes will be placed on the right median nerve (2 cm proximal to the wrist crease), with intensity set at 300% of the motor threshold, frequency at 10 Hz, pulse width at 1 ms, for a total duration of 120 min. The MNS will begin 10 min before tDCS starts and continue for 90 min after tDCS ends, ensuring a 20-min overlap of both stimulations (30). The combined tDCS and MNS stimulation protocol is shown in Figure 2, which details the stimulation parameters and timing sequence.

### Control group interventions

Control Group 1 (Sham tDCS+MNS group) will receive sham tDCS combined with MNS treatment. Sham tDCS will ramp up to 2 mA over 30 s, then ramp down to 0 over 30 s, and remain off thereafter (31). MNS parameters will be identical to the experimental group.

Control Group 2 (tDCS+Sham MNS group) will receive sham MNS combined with tDCS treatment. tDCS parameters will be identical to the experimental group, but MNS intensity will be set at 50% of the sensory threshold, insufficient to elicit muscle contraction (20).

### Permitted/prohibited concomitant interventions

All patients will continue to receive standard rehabilitation therapy, including physical and occupational therapy. Medications necessary for maintaining stable vital signs, such as antihypertensive and antiepileptic drugs, are permitted. The use of drugs that may significantly affect consciousness levels or brain excitability, such as high-dose sedatives or anesthetics, is prohibited (22). If such medications are required, their use will be documented and considered in the analysis. Participation in other experimental treatments or clinical trials is prohibited.

### Outcome measures

#### Primary outcome measure

The primary outcome measure is the change in total score on the Coma Recovery Scale-Revised (CRS-R) from baseline to post-treatment (24). The CRS-R is the gold standard for assessing patients with disorders of consciousness and comprises six subscales: auditory, visual, motor, oromotor, communication, and arousal, with a total score range of 0–23 (32). CRS-R assessments will be conducted by trained evaluators at baseline, after 4 weeks of treatment, and at 2 weeks, 4 weeks, 12 weeks, and 24 weeks post-intervention. To ensure consistency and accuracy, all evaluators will undergo standardized training and pass a video scoring test to ensure inter-rater reliability (ICC > 0.8) (24).

#### Secondary outcome measures

(1) Functional Near-Infrared Spectroscopy (fNIRS): To assess cortical hemodynamic changes, reflecting brain functional connectivity (33). Measurements will be taken at baseline and after 4 weeks of treatment.

(2) Electroencephalography (EEG): To evaluate changes in brain electrical activity, including power spectral analysis and functional connectivity analysis (34). Measurements will be taken at baseline and after 4 weeks of treatment.

(3) Event-Related Potentials (ERPs): To assess cognitive processing abilities, focusing on the P300 component (35). Measurements will be taken at baseline and after 4 weeks of treatment.

(4) Glasgow Outcome Scale-Extended (GOS-E): To evaluate overall functional outcomes (36). Assessments will be conducted at baseline, 4 weeks, and 12 weeks follow-up.

(5) Functional Magnetic Resonance Imaging (fMRI): To be assessed at baseline and 24 weeks follow-up.

(6) Adverse Events: All treatment-related adverse events will be recorded, including type, severity, and duration. Continuous monitoring will be conducted during each treatment session and throughout the follow-up period.

#### Predetermined visit schedule

The visit schedule for all assessments is presented in Table 1. Participants will be evaluated at screening (Week −2), baseline (Week 0), post-treatment (Week 4), and follow-up (Weeks 6, 8, 16, and 28). Demographic characteristics and medical history will be collected only at the first visit. Baseline assessments, including CRS-R, fNIRS, EEG, ERPs, GOS-E, and fMRI, will be conducted at Week 0, followed by randomization and treatment initiation. The primary outcome measure (CRS-R) will be assessed at all visits starting from baseline. Secondary outcome measures will be measured at various time points. All follow-up visits will be conducted in outpatient settings. For patients unable to attend in person, home visits or video follow-ups will be arranged (22).

**Table 1:** Schedule of enrolment, interventions, and assessments.

	Study Period						
	Screening	Baseline	Treatment	Follow-up			
Week	−2	0	4	6	8	16	28
Enrolment							
Eligibility screen	**×**						
Signed informed consent	**×**						
Allocation		**×**					
Randomization		**×**					
Interventions							
tDCS + MNS group		**×**	**×**				
tDCS sham + MNS group		**×**	**×**				
tDCS + MNS sham group		**×**	**×**				
Assessments							
Primary outcome							
CRS-R		**×**	**×**	**×**	**×**	**×**	**×**
Secondary outcomes							
fNIRS		**×**	**×**				
EEG		**×**	**×**				
ERPs		**×**	**×**				
GOS-E		**×**			**×**	**×**	
fMRI		**×**					**×**
Adverse Events			**×**	**×**	**×**	**×**	**×**

× Represents data collection timepoint. tDCS, transcranial direct current stimulation; MNS, median nerve stimulation; CRS-R, Coma Recovery Scale-Revised; fNIRS, Functional Near-Infrared Spectroscopy; EEG, Electroencephalography; ERPs, Event-Related Potentials; GOS-E, Glasgow Outcome Scale-Extended; fMRI, Functional magnetic resonance imaging.

#### Follow-up retention strategy

To minimize attrition during the 6-month follow-up period, we will implement several retention strategies: (1) Assigning a dedicated research coordinator to maintain regular contact with participants’ families/caregivers; (2) Providing flexible scheduling options for follow-up assessments, including the possibility of home visits when appropriate; (3) Reimbursing transportation expenses for hospital visits; (4) Implementing telephone or video follow-ups for interim check-ins between scheduled assessments; (5) Providing clear information about the importance of complete follow-up data; (6) Sending reminder communications before each scheduled assessment; and (7) Collecting multiple contact methods (phone, email, alternative contacts) to maintain communication if primary contact methods fail. Additionally, we will monitor reasons for withdrawal and address any systematic issues that emerge during the trial.

### Data collection and study safety

#### Data collection and quality control

This study will utilize an advanced electronic data capture (EDC) system for data management, employing the REDCap (Research Electronic Data Capture) platform (37). This system features real-time data validation, audit trails, and automated logic checks, significantly reducing data entry errors. All research personnel will receive specialized training on the REDCap system to ensure accuracy and consistency in data collection.

#### Study safety

Based on previous research, transcranial direct current stimulation and median nerve stimulation are considered relatively safe, non-invasive techniques (16). However, we will closely monitor potential adverse events, including but not limited to: (1) mild discomfort, redness, or itching at the stimulation site; (2) transient headache or dizziness; (3) nausea; (4) fatigue. To address these potential adverse events, we have developed a detailed response plan: (1) All research personnel will receive training in adverse event identification and management. (2) Given that patients with prolonged disorders of consciousness (pDoC) cannot reliably self-report subjective adverse events, a comprehensive monitoring approach has been implemented, including systematic observation of objective signs (skin reactions, autonomic changes such as heart rate, blood pressure, and sweating, facial expressions, and behavioral responses); physiological parameter monitoring (temperature, blood pressure, and heart rate before and after each session); neurophysiological indicators (EEG changes during treatment and follow-up that may indicate discomfort or adverse responses); caregiver reports (detailed inquiries to family members and caregivers about any unusual behaviors or responses); and clinical judgment (comprehensive assessment by experienced clinicians familiar with detecting subtle signs of discomfort in non-communicative patients). (3) Detailed assessments using the above methods will be conducted before and after each treatment session and at all follow-up visits. (4) Any identified adverse events will be documented and evaluated by a physician not involved in the direct treatment delivery. (5) For adverse events management, we will implement a standardized classification system based on the Common Terminology Criteria for Adverse Events (CTCAE v5.0). All adverse events will be graded on a 5-point scale (1: mild; 2: moderate; 3: severe; 4: life-threatening; 5: death-related) and systematically documented in a dedicated adverse event log. Events of grade 3 or higher will be reported to the ethics committee within 24 h.

Treatment will be immediately discontinued if any of the following criteria are met: (1) Grade 3 or higher adverse events related to the intervention; (2) Recurring grade 2 adverse events that persist despite mitigation measures; (3) Participant or legal representative request; (4) Investigator judgment that continuation poses unacceptable risk. The decision to discontinue treatment will be made by a physician not directly involved in intervention delivery, and all discontinuations will be reported to the ethics committee and study safety monitoring board within 48 h.

### Statistical analysis

#### Analysis principles

This study will employ intention-to-treat (ITT) analysis as the primary analytical approach to evaluate the efficacy of transcranial direct current stimulation combined with median nerve stimulation in patients with chronic disorders of consciousness (38). The ITT analysis will include all randomized patients, regardless of protocol adherence or treatment completion. Additionally, we will conduct a per-protocol (PP) analysis as a supplementary measure to assess treatment effects in patients who fully adhered to the study protocol (39).

#### Statistical methods

(1) Descriptive statistics: Continuous variables will be presented as mean ± standard deviation (for normally distributed data) or median and interquartile range (for non-normally distributed data); categorical variables will be presented as frequencies and percentages.

(2) Between-group comparisons: Baseline characteristics: Continuous variables will be compared using one-way analysis of variance (ANOVA) (for normally distributed data) or Kruskal-Wallis test (for non-normally distributed data); categorical variables will be compared using chi-square test or Fisher’s exact test.

(3) Primary outcome analysis: First, within-group comparisons will be conducted to assess changes in CRS-R scores from baseline to each follow-up time point within each intervention group. Paired t-tests (for normally distributed data) or Wilcoxon signed-rank tests (for non-normally distributed data) will be used for these comparisons. This will provide insights into the therapeutic effectiveness of each intervention approach. Subsequently, between-group comparisons will be performed using a 3 × 6 mixed repeated measures analysis of covariance (ANCOVA) with group (tDCS+MNS, sham tDCS+MNS, and tDCS+sham MNS) as a between-subjects factor and time (baseline, 4 weeks post-treatment, and follow-ups at 2 weeks, 4 weeks, 12 weeks, and 24 weeks) as a within-subjects factor. To control for potential confounding variables, baseline age, sex, duration of consciousness disorder, and baseline severity will be included as covariates if significant differences are observed at baseline despite randomization. Post-hoc analyses with appropriate multiple comparison corrections will be used to identify specific group differences at each time point. The analysis will include all assessment time points (baseline, 4 weeks post-treatment, and follow-ups at 2 weeks, 4 weeks, 12 weeks, and 24 weeks) (40). If data violate normality assumptions, generalized estimating equations (GEE) models will be employed (41).

(4) Secondary outcome analyses: For continuous secondary outcomes (e.g., fNIRS, EEG, and ERP metrics), both within-group and between-group analyses will be conducted following the same approach as the primary outcome. Within-group changes will be assessed first, followed by between-group comparisons with appropriate covariate adjustments. Categorical secondary outcomes (e.g., GOS-E) will be analyzed using ordinal logistic regression, with baseline measurements included as covariates to account for potential confounding.

(5) Missing data handling: Multiple imputation techniques will be used to handle missing data, reducing bias and improving statistical power, The imputation model will include all primary and secondary outcomes, as well as key baseline covariates (age, sex, time since injury, baseline CRS-R score, and consciousness state). We will generate 20 imputed datasets, as recommended by methodological guidelines (42); Results will be pooled using Rubin’s rules, and sensitivity analyses will include complete case analysis to assess the impact of missing data on results.

(6) Subgroup analyses: Pre-specified subgroup analyses will be conducted based on age, sex, duration of consciousness disorder, and baseline CRS-R scores to explore potential effect modifiers. To control for multiple comparisons in these subgroup analyses, we will apply the same Bonferroni correction approach used for secondary outcomes. Furthermore, we will clearly label these analyses as exploratory and interpret the results with appropriate caution regarding potential Type I error inflation.

(7) Sensitivity analyses: In addition to ITT analysis, PP analysis will be performed to evaluate treatment effects in patients who fully adhered to the protocol.

(8) Covariate handling: Despite our stratified randomization strategy, potential imbalances in baseline characteristics might still exist. Therefore, we will compare baseline demographic and clinical characteristics between groups. If any significant differences are detected (*p* < 0.05), these variables will be treated as covariates in subsequent analyses to minimize confounding. Additionally, baseline measurements of outcome variables will routinely be included as covariates in the respective analyses to account for potential regression to the mean effects and to increase statistical power.

All statistical analyses will be conducted using R software (version 4.1.0 or higher), with a significance level set at *α* = 0.05 (two-sided). To control for Type I error inflation due to multiple comparisons, Bonferroni correction will be applied to adjust *p*-values for secondary outcomes (43).

#### Ethical considerations

This study will strictly adhere to the principles outlined in the Declaration of Helsinki (44) and the Council for International Organizations of Medical Sciences (CIOMS) International Ethical Guidelines for Health-Related Research Involving Humans (45). The study protocol has been reviewed and approved by the Ethics Committee of Guangdong Sanjiu Brain Hospital (approval number: 2022–010-027) and registered with the Chinese Clinical Trial Registry (ChiCTR2300075190). We will consistently uphold ethical principles, ensuring autonomy, anonymity, confidentiality, and fairness throughout the research process. Written informed consent will be obtained from the legal representatives or relatives of all eligible participants. If a patient regains sufficient consciousness during the trial to comprehend the study information and provide informed consent, we will implement a re-consent procedure. This will involve providing age-appropriate and capacity-adjusted information about the study, assessing the patient’s comprehension, and obtaining their written consent to continue participation. The ethics committee will be notified of any such cases, and the patient’s decision will be respected, including the right to withdraw from the study, and any adverse events resulting from the intervention (although unlikely) will be addressed by the study. Participants may withdraw from the trial at any time. Any protocol amendments will be communicated to the ethics review committee.

## Discussion

pDoC following ICH severely impact patients’ quality of life and impose a significant societal burden (5). Despite some efficacy shown by existing treatments, notable limitations persist (10). In recent years, the combined application of tDCS and MNS has garnered attention due to its safety, ease of operation, and relatively low cost. Preliminary studies suggest potential benefits for patients with disorders of consciousness, but existing research has primarily focused on traumatic brain injury patients, with small sample sizes and short follow-up periods. Recent evidence indicates that 3–12 months post-ICH may be a critical period for neurological recovery, yet systematic studies targeting pDoC patients during this period are lacking. Given these research gaps and urgent clinical needs, we designed this prospective randomized controlled trial to investigate the therapeutic effects of tDCS combined with MNS in pDoC patients 3–12 months post-ICH. This study will conduct a 6-month follow-up, comprehensively assessing the long-term efficacy, safety, and potential mechanisms of this combined intervention. Through this research, we aim to provide more reliable and comprehensive scientific evidence for the clinical management of ICH-induced pDoC, paving the way for improving patient prognosis and quality of life.

Our study design has several notable advantages. Firstly, we focus on pDoC patients 3–12 months post-ICH, a period considered critical for neurological recovery. By targeting this specific population, we aim to obtain more precise and valuable data on treatment efficacy. Secondly, we employ a combined application of tDCS and MNS, a combination that may enhance therapeutic effects through synergistic action (46). tDCS modulates cortical excitability, while MNS stimulates the peripheral nervous system, potentially promoting more comprehensive neural network reorganization (47, 48). Thirdly, our 6-month long-term follow-up design will help evaluate sustained treatment effects and potential long-term safety issues. Lastly, we adopt multidimensional assessment metrics, including behavioral, electrophysiological, and neuroimaging indicators. This comprehensive assessment approach not only provides stronger evidence of therapeutic efficacy but may also reveal biomarkers of consciousness recovery in pDoC patients (49). This multifaceted evaluation strategy helps us better understand treatment effects and their underlying neural mechanisms.

The combined application of tDCS and MNS may promote consciousness recovery in pDoC patients through multiple mechanisms. Based on our stimulation sites (left dorsolateral prefrontal cortex at F3 position and right supraorbital area at Fp2 position), we have specific predictions regarding the neural effects and potential recovery mechanisms. tDCS may modulate cortical excitability by altering the resting membrane potential of neurons (13), potentially enhancing neuroplasticity and influencing neuronal activity through regulation of neurotransmitter release. We predict that anodal stimulation at the left DLPFC will enhance the excitability of this region, which plays a crucial role in executive functions and consciousness processing (50).

These mechanisms may contribute to the reconstruction of damaged neural networks and restoration of functional connectivity within specific brain circuits critical for consciousness (13, 51, 52). Specifically, we predict enhanced connectivity between: (1) left dorsolateral prefrontal cortex and bilateral thalamus, (2) left dorsolateral prefrontal cortex and posterior parietal cortex, and (3) medial prefrontal cortex and posterior cingulate cortex. These connectivity changes would reflect improved integration within the frontoparietal network and default mode network, both of which are often disrupted in pDoC patients (53, 54).

MNS, by stimulating the peripheral nervous system, can activate the ascending reticular activating system (ARAS) that maintains wakefulness and consciousness, while also promoting the release of neurotrophic factors (such as brain-derived neurotrophic factor, BDNF) that support neuronal survival and growth (55, 56). Through median nerve stimulation, we expect to observe increased activity in the somatosensory cortex, which may facilitate improved sensory processing and integration–key components for consciousness recovery (57).

The combined application of these two techniques may produce synergistic effects: tDCS-induced changes in cortical excitability may enhance the conduction and integration of MNS signals in the central nervous system, while MNS may enhance the effects of tDCS by increasing cortical responsiveness (48, 58). This synergy may more effectively promote the reorganization of brain functional networks (such as the default mode network and salience network) involved in maintaining consciousness (59). Furthermore, this combined therapy may act by modulating neuroinflammation and oxidative stress. Studies have shown that tDCS can reduce the expression of inflammatory factors, while MNS can regulate the autonomic nervous system, thereby influencing systemic inflammatory responses (60, 61). This anti-inflammatory action may help create a more favorable environment for neural repair. In summary, the combined application of tDCS and MNS may promote consciousness recovery in pDoC patients through multiple mechanisms, including modulating neuroplasticity, activating ARAS, promoting neurotrophic factors, reorganizing functional networks, and regulating neuroinflammation. This multi-target therapeutic strategy may be more effective than single interventions in addressing the complex pathophysiological characteristics of pDoC.

Our secondary outcome measures directly evaluate hypothesized mechanisms of tDCS+MNS effects. EEG analyses will assess cortical excitability and network reorganization in consciousness-critical frontoparietal and default mode networks. Event-related potentials (P300) will measure cognitive processing improvements. fNIRS will provide insights into vascular and metabolic changes, while fMRI will evaluate network connectivity changes, particularly in thalamocortical circuits and the ascending reticular activating system. By systematically analyzing these multiple physiological parameters, we can triangulate evidence to support or refute our mechanistic hypotheses regarding neuroplasticity, thalamocortical connectivity restoration, and anti-inflammatory effects.

Despite the numerous advantages of our study design, some limitations exist. Firstly, our study focuses only on pDoC patients following ICH, which may limit the applicability of our findings to pDoC patients with other etiologies. Secondly, although we implement a 6-month follow-up period, it may still be insufficient to comprehensively evaluate long-term treatment effects. Thirdly, while we adopt multidimensional assessment metrics, certain potential neurobiological changes may not be fully captured by existing technologies. Additionally, due to ethical considerations, we cannot implement a placebo control, which may affect the precise evaluation of treatment efficacy. Lastly, as a single-center study, we also recognize inherent limitations to the generalizability of our findings. Regional variations in standard care practices, rehabilitation protocols, and population demographics may influence treatment outcomes. Future multi-center trials across diverse geographical regions would be valuable to validate our findings and strengthen the evidence base for this intervention approach. Despite these limitations, we believe this study will provide valuable insights and data for the treatment of pDoC patients.
